# Ablação de Fibrilação Atrial por Radiofrequência de Alta Potência e Curta Duração: Técnica Ponto a Ponto ou de Arrasto de Cateter?

**DOI:** 10.36660/abc.20240771

**Published:** 2025-01-31

**Authors:** Guilherme Fenelon

**Affiliations:** 1 Hospital Israelita Albert Einstein Centro de Arritmia São Paulo SP Brasil Hospital Israelita Albert Einstein - Centro de Arritmia, São Paulo, SP – Brasil

**Keywords:** Ablação por Radiofrequência, Fibrilação Atrial, Veias Pulmonares

O isolamento das veias pulmonares (IVP) é universalmente aceito como a pedra angular da ablação da fibrilação atrial (FA). As diretrizes atuais^1^ recomendam o isolamento elétrico das veias pulmonares em todos os procedimentos de ablação da FA (Classe I, nível de evidência A). Atualmente, os métodos mais comuns para realizar IVP são a radiofrequência (RF) ponto a ponto guiada por mapeamento 3D ou o criobalão. Ambos são energias térmicas, criando lesões por aquecimento (RF) ou congelamento (crioenergia) do tecido. Vale ressaltar que foi consistentemente demonstrado que a ablação por RF e a ablação por criobalão são igualmente eficazes e seguras para tratar pacientes com FA paroxística. No entanto, a ablação por criobalão é mais reprodutível dentro dos grupos do que a ablação por RF.^2^ Os resultados da ablação por RF estão intimamente relacionados ao operador e ao volume do centro. Além disso, pode haver variabilidade significativa no número de lesões de ablação criadas, na força aplicada pela ponta do cateter ao tecido — conhecida como força de contato — e na quantidade e duração do fornecimento de energia.^1^

Os protocolos de ablação mudaram significativamente durante a última década, particularmente com relação aos parâmetros de RF. As configurações de potência convencionais eram de 20-35 W em 30-60 segundos. Nos últimos anos, os protocolos de alta potência e curta duração (HPSD) que utilizam RF em níveis de potência de 40-50 W para durações mais curtas ganharam popularidade.^3 - 6^ De fato, a técnica HPSD está associada a tempos mais curtos para atingir IVP e maior liberdade de recorrências de FA, embora com uma tendência para mais êmbolos cerebrais assintomáticos.^4 , 5^

O objetivo da ablação de FA com energia de RF é criar lesões circunferenciais contínuas ao redor do antro das veias pulmonares.^1^ A RF pode ser administrada por meio de uma técnica ponto a ponto, na qual aplicações únicas são criadas em cada ponto antes de mover a ponta do cateter para o próximo ponto. Embora altamente eficaz, essa abordagem pode ser demorada. Para superar essa limitação, uma técnica de arrasto foi desenvolvida.^1 , 7^ Nesse método, a RF é administrada continuamente à medida que o cateter é arrastado ao longo da linha de lesão pretendida. Esse método encurta a duração do procedimento e tem sido amplamente utilizado, mas com a introdução de marcadores avançados de qualidade de lesão em tempo real,^4 , 8^ ele perdeu a popularidade.^7^

A durabilidade do IVP é o fator mais importante que afeta as taxas de recorrência de FA.^1^ Assim, para obter IVP durável, é fundamental criar lesões de ablação de boa qualidade. Os sistemas atuais de ablação por RF integraram algoritmos de predição de lesões em tempo real para estimar as características e a qualidade das lesões. O índice de ablação (AI) incorpora força de contato, tempo e potência em uma fórmula ponderada, enquanto o índice de lesão (IL) usa tempo, potência, força de contato e impedância.^1 , 4 , 8^ As estratégias de ablação guiadas por AI e IL demonstraram melhorar os resultados do procedimento, além de reduzir a duração do procedimento e o tempo de RF.^1 , 4 , 8^ Como resultado, os indicadores de qualidade da lesão tornaram-se regularmente usados pela maioria dos eletrofisiologistas em todo o mundo. No entanto, é importante observar que a ablação guiada por AI e IL é limitada a técnicas ponto a ponto.^4 , 7 , 8^

Nesta edição dos ABC, Vassalo et al.^9^ relatam 214 pacientes (idade média de 61 ± 12 anos) submetidos à ablação de FA usando HPSD (50 W) com uma técnica de arrasto na qual o cateter era movido a cada 2-5 segundos durante a administração contínua de RF. A maioria dos pacientes era do sexo masculino (67%) com FA paroxística (58%). Além disso, as dimensões atriais e a fração de ejeção do ventrículo esquerdo eram normais. Em um acompanhamento médio de 32,8 ± 13,2 meses, a recorrência de FA foi observada em 43 pacientes (20,1%). Como esperado, as taxas de recorrência foram maiores na FA persistente (26,7%) em comparação à FA paroxística (15,3%). A análise multivariada identificou os seguintes preditores clínicos de recorrência: idade ≥ 65 anos (p= 0,006); obesidade [índice de massa corporal > 30 (p= 0,009)]; Pontuação CHA2DS2VASC ≥ 3 (p= 0,003) e FA persistente (p= 0,045). Além disso, um aumento na frequência cardíaca ≥ 30% (p= 0,04) e < 60 min no átrio esquerdo (p= 0,007) foram preditores de sucesso da ablação. Infelizmente, as recorrências de FA foram avaliadas com ECGs e gravações de Holter de 24 horas, o que pode ter superestimado as taxas de sucesso. Além disso, 73 de 100 (73%) pacientes submetidos à cardioversão elétrica no dia da ablação não converteram para ritmo sinusal e foram excluídos da análise. Portanto, os achados do estudo em pacientes com FA persistente devem ser vistos com cautela.

Apesar dessas limitações, os resultados do estudo são comparáveis aos ensaios de ablação controlada usando HPSD e abordagem de indicadores guiados por lesão em pacientes com FA paroxística com corações normais.^4 - 6^ Além disso, todos os preditores clínicos de recorrência estão bem estabelecidos na literatura.^10^ A significância dos aumentos na frequência cardíaca e menor tempo de permanência no átrio esquerdo como preditores do sucesso da ablação é menos clara. O primeiro pode refletir denervação vagal, mas inflamação e medicamentos antiarrítmicos também afetam a frequência cardíaca.^1^ O último pode ser simplesmente um marcador de uma anatomia atrial esquerda amigável. Tomadas em conjunto, essas observações sugerem que HPSD usando a técnica de arrasto ou abordagem ponto a ponto guiada por IA e IL pode produzir resultados semelhantes. Na ausência de grandes ensaios clínicos randomizados comparando essas técnicas,^1 , 7^ a escolha entre ablação por arrasto ou ponto a ponto deve considerar a preferência e a experiência do operador.

O estudo atual destaca a heterogeneidade marcante dos protocolos de ablação por RF empregados para atingir IVP.^1 , 2^ Apoiando essa premissa, o HPSD foi realizado usando 40 W, 45 W ou 50 W.^3 - 6^ Embora o arrasto do cateter ainda seja usado por alguns grupos, é improvável que se utilize a mesma abordagem de ablação e configurações de RF que Vassalo et al.^9^ O mesmo vale para a técnica de escolha indiscutível de IA e ablação ponto a ponto guiada por IL, na qual a personalização dos parâmetros de RF e conjuntos de lesões ocorre com frequência.^4 , 8^ Portanto, é importante reconhecer que a abordagem de ablação ideal e as configurações de RF em termos de segurança e eficácia ainda precisam ser determinadas. Por fim, no final das contas, independentemente da técnica usada, o que realmente importa é obter IVP de forma consistente.
