# Supplementary material for: Ablação de Fibrilação Atrial por Radiofrequência de Alta Potência e Curta Duração: Técnica Ponto a Ponto ou de Arrasto de Cateter?
Source: Arq Bras Cardiol. 2025 Jan 31;121(12):e20240771. [Article in Portuguese] doi: 10.36660/abc.20240771 (PMC12092023; doi:10.36660/abc.20240771)
Supplement: Translation

# Translation: High-power, Short-duration Radiofrequency Ablation of Atrial Fibrillation: Point-by-Point or Catheter Dragging Technique?

**DOI:** 10.36660/abc.20240771i

**Authors:** Guilherme Fenelon

**Affiliations:** 1 São Paulo SP Brazil Hospital Israelita Albert Einstein - Centro de Arritmia, São Paulo, SP – Brazil

**Keywords:** Radiofrequency Ablation, Atrial Fibrillation, Pulmonary Veins

Pulmonary vein isolation (PVI) is universally accepted as the cornerstone of atrial fibrillation (AF) ablation. Current guidelines^1^recommend electrical isolation of the pulmonary veins in all AF ablation procedures (Class I, level of evidence A). As of today, the most common methods to perform PVI are point-by-point radiofrequency (RF) guided by 3D mapping or the cryoballoon. Both are thermal energies, creating lesions by heating (RF) or freezing (cryoenergy) the tissue. Of note, it has been consistently demonstrated that RF ablation and cryoballoon ablation are equally effective and safe for treating patients with paroxysmal AF. However, cryoballoon ablation is more reproducible within groups than RF ablation.^2^The outcomes of RF ablation are closely related to operator and center volumes. Furthermore, there can be significant variability in the number of ablation lesions created, the force applied by the tip of the catheter to the tissue—known as contact force—and the amount and duration of power delivery.^1^

Ablation protocols have changed significantly during the last decade, particularly with respect to RF parameters. Conventional power settings were 20-35 W over 30-60 seconds. In recent years, high-power, short-duration (HPSD) protocols utilizing RF at power levels of 40-50 W for shorter durations have gained popularity.^3 - 6^Indeed, the HPSD technique is associated with shorter times to achieve PVI and greater freedom from AF recurrences, albeit with a trend towards more asymptomatic cerebral emboli.^4 , 5^

The goal of AF ablation with RF energy is to create continuous circumferential lesions around the antra of the pulmonary veins.^1^RF can be delivered via a point-by-point technique, in which single applications are created at each point before moving the catheter tip to the next point. Although highly effective, this approach may be time-consuming. To overcome this limitation, a dragging technique has been developed.^1 , 7^In this method, RF is continuously delivered as the catheter is dragged along the intended lesion line. This method shortens the procedural duration and has been widely used, but with the introduction of advanced real-time lesion quality markers,^4 , 8^it has lost favor.^7^

The durability of PVI is the most important factor affecting AF-recurrence rates.^1^Thus, to obtain durable PVI, it is paramount to create good-quality ablation lesions. Current RF ablation systems have integrated real-time lesion prediction algorithms to estimate lesion characteristics and quality. The ablation index (AI) incorporates contact force, time, and power in a weighted formula, whereas the lesion index (LSI) uses time, power, contact force, and impedance.^1 , 4 , 8^AI and LSI-guided ablation strategies have been shown to improve procedural outcomes in addition to reducing procedure duration and RF time.^1 , 4 , 8^As a result, lesion quality indicators have become regularly used by most electrophysiologists worldwide. However, it is important to remark that AI and LSI guided ablation are limited to point-by-point techniques.^4 , 7 , 8^

In this issue of the ABC, Vassalo et al.^9^report on 214 patients (mean age 61±12 years) undergoing AF ablation using HPSD (50 W) with a dragging technique in which the catheter was moved every 2-5 seconds during continuous RF delivery. Most patients were male (67%) with paroxysmal AF (58%). Further, atrial dimensions and left ventricular ejection fraction were normal. At a mean follow-up of 32.8±13.2 months, AF recurrence was observed in 43 patients (20.1%). As expected, recurrence rates were higher in persistent AF (26.7%) as compared to paroxysmal AF (15.3%). Multivariate analysis identified the following clinical predictors of recurrence: age ≥ 65 years (p= 0.006); obesity [body mass index > 30 (p= 0.009)]; CHA2DS2VASC score ≥ 3 (p= 0.003) and persistent AF (p= 0.045). In addition, an increase in heart rate ≥ 30% (p= 0.04) and < 60 min in the left atrium (p= 0.007) were predictors of ablation success. Unfortunately, AF recurrences were assessed with ECGs and 24-hour Holter recordings, which may have overestimated success rates. Further, 73 out of 100 (73%) patients undergoing electrical cardioversion on the day of ablation did not convert to sinus rhythm and were excluded from the analysis. Therefore, the study findings on persistent AF patients should be viewed with caution.

Notwithstanding these limitations, the study results are comparable to controlled ablation trials using HPSD and lesion guided indicators approach in paroxysmal AF patients with normal hearts.^4 - 6^Furthermore, all clinical predictors of recurrence are well-established in the literature.^10^The significance of increases in heart rate and shorter left atrium dwell time as predictors of ablation success is less clear. The former may reflect vagal denervation, but inflammation and antiarrhythmic drugs also affect heart rate.^1^The latter may simply be a marker of a friendly left atrial anatomy. Taken together, these observations suggest that HPSD using the dragging technique or AI and LSI-guided point-by-point approach may yield similar outcomes. In the absence of large randomized controlled trials comparing these techniques,^1 , 7^the choice between dragging or point-by-point ablation should consider operator preference and experience.

The current study highlights the marked heterogeneity of RF ablation protocols employed to achieve PVI.^1 , 2^Supporting this premise, HPSD has been performed using either 40 W, 45 W, or 50 W.^3 - 6^Although catheter dragging is still used by some groups, it is unlikely that one utilizes the same ablation approach and RF settings as Vassalo et al.^9^The same holds for the indisputable top pick technique AI and LSI-guided point-by-point ablation in which personalization of RF parameters and lesion sets frequently occur.^4 , 8^Hence, it is important to acknowledge that the optimal ablation approach and RF settings in terms of safety and efficacy remain to be determined. Lastly, at the end of the day, regardless of the technique you use, what really matters is to obtain PVI consistently.
